# Prevalence of Vaccine Type Infections in Vaccinated and Non-Vaccinated Young Women: HPV-IMPACT, a Self-Sampling Study

**DOI:** 10.3390/ijerph15071447

**Published:** 2018-07-09

**Authors:** Emilien Jeannot, Manuella Viviano, Charlotte de Pree, Mona Amadane, Emmanuel Kabengele, Pierre Vassilakos, Patrick Petignat

**Affiliations:** 1 Institute of Global Health-Faculty of Medicine, Chemin de Mines 9, 1202 Geneva, Switzerland; Emmanuel.Kabengele@unige.ch; 2 Community Psychiatric Service, Lausanne University Hospital (CHUV), 1011 Lausanne, Switzerland; 3 Gynecology Division, Department of Obstetrics and Gynecology, Geneva University Hospitals, Boulevard de la Cluse 30, 1205 Geneva, Switzerland; manuela.viviano@hcuge.ch (M.V.); patrick.petignat@hcuge.ch (P.P.); 4 Faculty of Medicine, University of Geneva, 1205 Geneva, Switzerland; Charlotte.De-Pree@etu.unige.ch (C.d.P.); mona.amadane@etu.unige.ch (M.A.); 5 Geneva Foundation for Medical Education and Research, Route de Ferney 150, 1211 Geneva 2, Switzerland; pierrevassilakos@bluewin.ch

**Keywords:** HPV, self-sampling, vaccination

## Abstract

**Background:** The human papillomavirus (HPV) vaccination program for young girls aged 11–26 years was introduced in Switzerland in 2008. The objective of this study was to evaluate the prevalence of high- and low-risk HPV in a population of undergraduate students using self-sampling for monitoring the HPV vaccination program’s effect. **Methods:** Undergraduate women aged between 18–31 years, attending the Medical School and University of Applied Sciences in Geneva, were invited to participate in the study. Included women were asked to perform vaginal self-sampling for HPV testing using a dry cotton swab. **Results:** A total of 409 students participated in the study—aged 18–31 years—of which 69% of the participants were vaccinated with Gardasil HPV vaccine and 31% did not received the vaccine. About HPV prevalence, 7.2% of unvaccinated women were HPV 16 or 18 positive, while 1.1% of vaccinated women were infected by HPV 16 or 18 (*p* < 0.01). Prevalence of HPV 6 and 11 was 8.3% in non-vaccinated women versus 2.1% in vaccinated women (*p* < 0.02). We observed no cross-protection for the other HPV genotypes of a low- and high-risk strain. **Conclusions:** Prevalence of HPV 6/11/16/18 was lower in vaccinated women versus unvaccinated women. Continued assessment of HPV vaccine effectiveness in real population is needed.

## 1. Introduction

Cervical cancer is one of the leading causes of cancer-related death among women worldwide [1]. The development of cervical precancerous and cancerous lesions is a direct consequence of genital human papillomavirus (HPV) infection, which has been identified as the most common sexually transmitted infection in the world [2]. The introduction of HPV vaccinations represents a primary preventive measure, which, if given to young girls prior to the onset of sexual activity, can potentially alleviate the burden of the HPV infection [3]. Recent studies have predicted that cervical cancer rates will be drastically reduced in about 10–15 years, thanks to the impact of the HPV vaccination and HPV-based screening [4].

In the United States, where the HPV vaccination was introduced in 2006, the population-based sentinel surveillance system has shown that the prevalence of HPV-16/18 in cervical intra-epithelial neoplasia grade 2 or worse (CIN2+) has decreased from 53.6% to 28.4% among women who have received at least one dose of the vaccine [5]. Another trial conducted in England has found HPV-16/18 prevalence to be reduced from 19.1% to 6.5% prior to and after the introduction of the vaccine, respectively [6]. In Australia, monitoring surveillance demonstrated a very low prevalence of vaccine-related HPV genotypes after eight years post-initiation of a national HPV vaccination program [7].

About 300 women in Switzerland are diagnosed with cervical cancer annually, with a risk of 2 per 100 of dying from this disease. The HPV vaccination program for young girls aged 11–26 years was launched in Switzerland in 2008 as a part of cervical cancer prevention, with the aim to prevent cervical cancer and other HPV-related disease. The quadrivalent vaccine (targeting HPV16, 18, 6, and 11) is currently administered to girls aged 11–14 years, both in schools and in healthcare centers. While it is known that the vaccination coverage rate varies widely among the different Swiss cantons, from a minimum of 20% to as much as 60% of the target population, little is known about the vaccination’s direct impact on the HPV infection rates. The differences in cantonal coverage rates can be explained by the fact that each Swiss canton organizes the vaccination campaigns and the relative program on its own, thus explaining the disparities and the lack of national coordination [8]. The lack of current data on the impact of the HPV vaccination in the country, therefore, makes it difficult to monitor the program’s efficacy.

The primary aim of this study was to evaluate the prevalence of high- and low-risk HPV in a population of undergraduate students using self-sampling for HPV testing. The results of this study will allow an estimation of the HPV vaccination program’s effectiveness, as well as the acceptability of HPV self-sampling as a means to track down the infection among vaccinated young girls in Switzerland.

## 2. Methods

### 2.1. Study Population and Setting

This study took place in the city of Geneva, which is situated in the canton of Geneva, Switzerland, between January 2016 and October 2017. The enrolled participants were undergraduate nurse and midwife students in their first, second, or third year of studies, as well as undergraduate students attending their first through fifth year of Medical School at the University of Geneva (years 1 to 6). All women aged 18–31 years were included; exclusion criteria were history of total hysterectomy or having undergone cervical treatment in the past 12 months.

### 2.2. Study Procedure

Information about the trial was delivered through the University website and by the study investigators, who sent an email to the target population describing the study and then delivered a short presentation about the study after the main course’s classes.

The HPV self-collection kit was directly distributed to women who expressed an interest to participate in the study at the end of class. The kit included a dry Dacron swab; a collection tube; instructions with explanatory pictures for self-sampling; a flyer explanation about HPV infection, cervical cancer screening, and cervical cancer; an informed consent form; and a questionnaire on socio-demographics. Self-sampling was performed at home, and the kit, including both the swab and filled-out questionnaire, was collected by the study investigators two to three days later. Additional information about HPV and the test results were delivered by a designated study investigator upon request. Sampling kits were provided free of charge.

### 2.3. Data Collection

Each participant was asked to fill out a questionnaire reporting her socio-demographic characteristics (age, nationally), sexual behaviors (number of sexual relations, use of contraception/protection device), questions about HPV vaccination (number of doses received, name of the vaccine), and questions about her acceptability of self-sampling.

### 2.4. Self-Sampling Procedure and Sample Preparation

Women were asked to gently insert the swab in the vagina, while being careful to avoid contact with the external genitalia, and to carefully turn it up to five times either clockwise or counter-clockwise. They were asked to then place the swab back into the dry tube, and to securely close it and put it back into its plastic bag containing the rest of the kit’s material.

Each swab was then placed into a tube containing 3 mL of ThinPrep and vortexed for 45 s. A total of 350 µL of the solution was then placed into a 5-mL, cone-shaped bottom tube (Eppendorf Tube, Merck KGaA, Darmstadt, Germany). The samples were promptly sent to Buhlmann laboratories for analysis.

### 2.5. Laboratory Analysis

DNA extraction was performed using the NIMBUS-IVD (Hamilton, Reno, Nevada) and the extraction reagents StarMag (Seegene, Seoul, Korea). Amplification and detection was then performed with the Anyplex™ II HPV high risk (HR) Detection (Seegene, Seoul, Korea) using the CFX96™ real-time thermocycler. Data recording and interpretation were automated. Anyplex II is a semi-quantitative real-time multiplex PCR assay for screening and HPV genotyping. This test uses dual priming oligonucleotides (DPO™) and tagging oligonucleotide cleavage and extension (TOCE™) technologies and allows the simultaneous detection and genotyping of 19 high-risk HPVs (including types 16, 18, 26, 31, 33, 35, 39, 45, 51, 52, 53, 56, 58, 59, 66, 68, 69, 73, and 82) and 9 low-risk HPVs (6, 11, 40, 42, 43, 44, 54, 61, and 70). As an internal control of assay validity, the β-globin gene is also detected. By knowing the step at which the melting curve becomes positive, semi-quantification of the DNA load of the β-globin and HPV genomes is made possible; this can vary from low (+; positive after 40 PCR cycles, <10^2^ copies/reaction), to intermediate (++; positive within 31 to 39 PCR cycles, ≥10^2^ and <10^5^ copies/reaction), to high (+++; positive before 31 PCR cycles, ≥10^5^ copies/reaction).

Whenever the quantity of HPV genome was not high enough to be detected by the Anyplex II device after running up to 40 PCR cycles, the test result was considered invalid. Analyses were run twice before considering the test result as “invalid”.

### 2.6. Study Sample

Sample size was obtained based on estimated prevalence of 6% of HPV 16/18 infection in the Swiss population aged less than 30 years. A total of 400 specimens would be needed to detect about an 85% reduction in HPV 16/18 prevalence (prevalence of 0.9% in the vaccinated population), given an 80% power and a two-sided significance level of 95%. Therefore, we estimate that a sample size of 400 women will be adequate for the analyses.

### 2.7. Statistical Analyses

Statistical analyses were run using STATA 13. Normality of the distribution was tested by the Kolmogorov–Smirnov test. Descriptive statistics and frequencies were analysed for all variables. Prevalence of HPV infections was evaluated for any HPV type, low vaccinated types (6 and 11), and high-risk types (16 and 18). Low- and high-risk non-vaccine types were also evaluated. *t*-test and Chi square test were used for the descriptive statistics and for the comparison between variables. A *p* value of less than 0.05 was considered as statistically significant.

A multivariate logistic regression was used to compare differences in HPV prevalence between vaccinated and unvaccinated women. It was also performed to identify factors associated with HPV prevalence and socio-demographic factors. The status of HPV infection (infected or not infected) was used as the primary outcome. In multivariable models, only those covariates that were of a priori interest of univariate analysis were included.

### 2.8. Ethical Approval

The study was approved by the Central Ethics Committee on Human Research of the Geneva University Hospitals (approval number: 15-257). This study was conducted in accordance with the Swiss law, as well as in accordance with the recommendations of Good Clinical Practices (ICH E6-1996) and the Declaration of Helsinki (Fortaleza, Brazil, October 2013). The trial was registered under cliniclatrials.gov with the identifiers: NCT03474211.

## 3. Results

### 3.1. Participants’ Socio-Demographic Characteristics

A total of 409 undergraduate students performed HPV self-sampling at home for HPV DNA testing and filled out the given questionnaire, and were thus included in the study.

The participants’ baseline characteristics are presented in Table 1. The mean age was 24 years (range 18–27); 55% of the participants were medical school undergraduate students (first to sixth year of medical curriculum), the other 45% were nursing students or midwives in their first, second, or third year of their Bachelor’s degree). The majority of the participants were Swiss (89%), 7% of them came from France, and 3% came from other European countries or non-European countries (South America or Africa).

A total of 80% of the participants were non-smokers and 8.6% smoked on a daily basis. Overall, 2.4% of the women reported never having had sexual intercourse; these women were nevertheless included in data analyses.

### 3.2. Vaccination Status

Overall, 69.4% of the participants were vaccinated with a minimum of one dose (284/409). Among the vaccinated participants, 72% had received all three doses of the HPV vaccine, while 21% had received two doses and 7% had received only one dose. All participants in our study were vaccinated with Gardasil.

A total of 75% of the vaccinated students were aged 18–23 years; the vaccination coverage rate was not statistically different between the medical students and the nursing students or midwives (71% versus 67%, respectively, *p* = NS). The mean age at the time of first vaccination dose was 14.8 years. The majority of the participants (75%) reported that the HPV vaccination was as important as the other vaccinations recommended in Switzerland, while up to 18% of them believed that this vaccination was less important than other vaccinations recommended in Switzerland.

### 3.3. HPV Genotype Prevalence and Distributions

**Figure 1:**
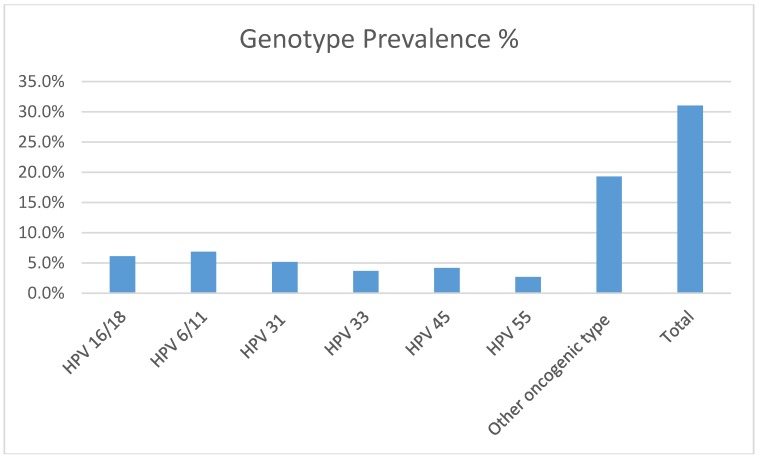
Human papillomavirus (HPV) genotype prevalence and distributions. In the case of multiple genotypes, each genotype was counted independently.

Figure 1 shows the genotype and prevalence distribution of HPV infection according to the genotype. Overall, 31.1% (127/409) of the swabs were positive for the presence of HPV DNA. Gardasil-targeted HPV genotypes were detected in 6.1% of the participants, who were positive for HPV-16/18, while 6.8% of the women were positive for HPV-6/11. A total of 15% of the participants were infected by multiple HPV genotypes. The prevalence of other HPV genotypes was 5.1 for HPV-31, 3.7% for HPV-33, 4.2% for HPV-45, and 2.7% for HPV-55.

**Figure 2:**
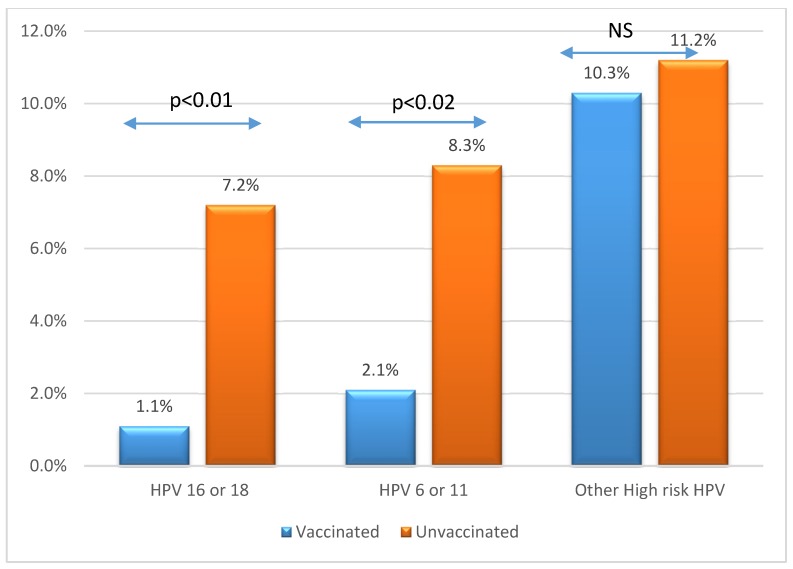
HPV prevalence 6/11 and 16/18, and other high-risk HPV according to vaccination status.

Figure 2 presents the HPV prevalence of Gardasil-targeted genotypes; we found that 7.2% of the unvaccinated population was HPV-16/18-positive, while only 1.1% of vaccinated women were infected by HPV-16/18 (*p* < 0.001). The prevalence HPV-6/11 was 8.3% among unvaccinated women versus 2.1% in the vaccinated group (*p* < 0.02). This difference was statistically significant for women of all ages. Prevalence for other HPV high-risk strains was not statistically different between vaccinated and unvaccinated women: 10.3% versus 11.2% *p* = NS.

### 3.4. Relationship between HPV Infection and Sociodemographic

Table 2 reports the association between country of origin and HPV positivity. Non-Swiss women had higher odds ratios of being HPV-positive than the participants who came from Switzerland, (Adjusted OR (aOR) = 4.4 confidence interval (CI) 95% [1.3–7.6] and aOR = 3.8 CI 95% [2.4–4.1], respectively). There was also a very strong association between sexual activity and HPV positivity, as young women who reported having more than five different sexual partners throughout their sex life had higher odds of being infected with HPV when compared with women who had only one sexual partner aOR = 7.8 CI 95% [2.4–12.2]. Female students who sometimes used condoms were more likely to be HPV-infected than those who reported always using condoms aOR 7.5 CI 95% [6.3–8.7], this relationship was also found for those who never used a condom aOR 6.6 CI 95 % [4.8–8.2].

### 3.5. Acceptability of Self-Sampling

Overall, 100% of the participants accepted to repeat self-sampling in order to evaluate their HPV clearance over time, and 85% of the participants reported that they would prefer self-sampling to the conventional pap smear for cervical cancer screening in the near future (*p* = 0.001). A total of 76% participants reported that self-sampling was not painful; only 8% found self-sampling very painful, while 97% found that self-sampling was easy to use.

## 4. Discussion

In this analysis performed on self-collected home samples of undergraduate medical and non-medical students, we found a low HPV prevalence of the Gardasil-targeted HPV genotypes. The canton of Geneva vaunts one of the best immunization coverage rates in Switzerland, reaching a target population coverage of nearly 80% [9]. One can assume that our results would have been very different in a canton with low immunization rates, which can go as low as less than 20% of the target population.

Our population constituted of future medical doctors, midwives, and nurses, and had a lower vaccination rate than that of the general population [9]. This under-representation of the vaccination rate among health professionals has been observed for other vaccines as well, such as the influenza vaccine [10,11,12].

We found a statistically significant difference in the prevalence of high-risk genotypes (6/11/16 and 18) between vaccinated and unvaccinated young women. These findings confirm the results of other studies on the effectiveness of the HPV vaccination as a means to decrease the prevalence of vaccine-targeted HPV types [13,14,15,16]. On the other hand, we observed no cross-protection for the other HPV genotypes, as we found no significant difference in the prevalence of non-Gardasil targeted genotypes between vaccinated and unvaccinated women, similar to other studies on the subject [17,18].

Other studies, however, have questioned this non-cross-protection. Saccucci et al. have shown a cross-protection in the first eight years after the HPV vaccine’s introduction in the United States in 2006 [19]. Another study assessing the effect of the introduction of the vaccine on the rates of infection of non-vaccine HPV genotypes in community settings have demonstrated a possible cross-protection effect [20,21], although the clinical significance of such phenomena is not yet fully understood, nor is it sufficiently evidence-based to draw conclusions. Continuous monitoring of HPV genotypes, both vaccine-targeted and non-vaccine-targeted, is important to evaluate the possible cross-protection effect. It is possible that, with the forthcoming of the nine-valent HPV vaccine in Switzerland, the prevalence of other HPV genotypes in the population will drop.

Our results support the existence of associations between country of birth and number of sexual partners with the likelihood of HPV infection. To reduce the impact of these risk factors on the development of the relative sexually-transmitted infection, public health campaigns should be directed toward promoting a greater population awareness about the HPV infection’s transmission, outcomes, and primary measures of prevention.

The use of self-sampling to measure the prevalence, distribution of HPV genotypes, and HPV vaccination effectiveness in our study population has proven to be effective. Moreover, a meta-analysis on the subject has shown that when PCR-based assays that amplify DNA viral sequences are used, the performance of HPV testing on clinician-collected samples is comparable to that of self-collected samples, such as the ones used in the present study [22]. Self-sampling has been reported to be more acceptable than physician-performed cytology testing, with women describing self-sampling as far more comfortable and practical than clinician-based sampling, which systematically entails a pelvic examination [23]. In our study, self-sampling proved to be a valid alternative to the standard vaccination program monitoring, thus proving to be a rather promising public health tool to monitor the effectiveness of HPV vaccination programs. Similarly, another study conducted in Canada has found that this strategy was a valid alternative to physician-performed vaginal sampling to evaluate the effectiveness of the HPV vaccination program [24,25].

## 5. Strength and Limitations

To our knowledge, this study was one of the first to directly assess the prevalence of HPV and the effectiveness of the HPV vaccination directly in the population through the use of self-sampling in Switzerland. The other studies carried out on the subject had a more modeling objective of the prevalence of this infection after the introduction of the HPV vaccination in Switzerland without trying to measure it directly in the population [26], but one large study with another methodology had shown the same results in another Swiss county [27].

Another strength was represented by the fact that we used a real-time PCR to estimate the HPV prevalence in the study population. In addition, as opposed to other trials using self-sampling, which registered between 0.5 and 0.7% of unsatisfactory HPV test results, we had no invalid results.

This study has some limitations that need to be addressed. The population sample is constituted exclusively of undergraduate students, which limits the generalization of our findings to other populations or settings. Additionally, the study sample size was not powered to detect any potential cross-protection of the vaccine-targeted HPV genotypes.

## 6. Conclusions

Our findings support the HPV vaccination’s effectiveness as a means to lower the prevalence of the infection with most oncogenic genotypes in a population of young women. The decreasing prevalence of the infection, therefore, represents one step closer to the prevention of the development of cervical cancer, which is the vaccination’s long-term aim. As self-sampling was well accepted by participants for monitoring the effectiveness of the HPV vaccination program, such a finding may support the use of self-sampling for cervical cancer screening, in the view of alleviating the world population from the burden of cervical cancer. In this study, we observed no cross-protection for the other HPV genotypes—low- and high-risk strains—between vaccinated and unvaccinated women.

## Figures and Tables

**Table 1:** Study participants socio-characteristics. HPV—human papillomavirus.

Characteristic	Study Population (*n* = 409)			
	*n*	% or Mean	95% Confidence Interval	
**Age (mean years)**		24	21.1	27.2
**Recruitment site**				
Faculty of medicine	225	55.0%	50.2	59.8
School of health sciences	184	45.0%	40.2	49.8
**Country of birth**				
Switzerland	365	89.2%	85.9	92
France	30	7.3%	5.1	10.2
Other European country	10	2.4%	1.2	4.3
other country	4	1.0%	0.3	2.3
**Tobacco smoking**				
Yes, every day	35	8.6%	6.1	11.6
Yes, but not every day	45	11.0%	8.2	14.3
No, never	329	80.4%	76.4	84
**Have you ever had sexual intercourse**				
Yes	399	97.6%	95.7	98.7
No	10	2.4%	1.2	4.3
**Your age at your first sexual intercourse (mean years)**				
Average	17		16.7	17.2
**How many sexual partners did you have in your life (mean number of partner)**				
Average	5.3		4.5	6.2
**Do you use condoms as a means of protection/contraception**				
Never	120	29.3%	22.7	31.4
Sometimes	90	22.0%	18.2	26.2
Often	100	24.4%	20.5	28.8
Always	99	24.2%	20.2	28.4
**Have you been vaccinated against HPV**				
Yes	284	69.4%	64.8	73.7
No	125	30.6%	26.4	35.2
**How many doses of HPV vaccine have you received (only for vaccinated women *n* = 284)**				
One	20	7.0%	7.4	14.5
two	60	21.1%	16.7	26.2
Three	204	71.8%	66.4	76.8
**Your age when you receive the first dose of the HPV vaccine (mean years)**				
Average	14.8		14.2	15.6
**Have you checked your vaccination record to answer previous questions**				
Yes	220	53.8%	49	58.6
No	189	46.2%	41.4	51.1
**In general, do you think that HPV vaccination is a vaccination:**				
More important than others	14	3.4%	1.9	5.5
Less important than others	74	18.1%	14.6	22
As important as the others	321	78.5%	74.3	82.3
**Would you recommend to your family/friends this self-sampling as monitoring vaccination**				
Yes	367	89.7%	86.5	92.4
No	42	10.3%	7.6	13.5
**In case of positivity of your self-collection, we authorize you to contact you again**				
Yes	405	99.0%	97.6	99.7
No	4	1.0%	0.3	2.3

**Table 2:** Association between HPV positivity and socio demographic factor.

	OR	95% CI	Adjusted OR	95% CI
**Recruitment site**				
Faculty of medicine	1	-	1	-
School of health sciences	1.3	0.9–1.7	1.4	0.9–1.8
**Country of birth**				
Switzerland	1	-	1	-
France	1.2	0.5–1.9	0.9	0.3–1.6
Other European country	**3.4**	**2.1–3.7**	**3.8**	**2.4–4.1**
other country	**4.9**	**1.7–8.1**	**4.4**	**1.3–7.6**
**Tobacco smoking**				
yes, every day	1.7	0.7–2.7	1.5	0.5–3.1
yes, but not every day	1.5	0.8–2.7	1.7	0.9–2.8
No, never	1	-	1	-
**Have you ever had sexual intercourse**				
Yes	**7.8**	**6.7–8.9**	**7.2**	**6.2–8.5**
No	1	-	1	-
**How many sexual partners did you have in your life (mean number of partner)**				
**0**	0.2	0.01–0.35	0.3	0.01–0.37
**1**	1	-	1	-
**2–5**	**3.6**	**2.1–6**	**3.3**	**2.3–6.3**
**>5**	**9.8**	**5.4–14.2**	**7.8**	**2.4–12.2**
**Do you use condoms as a means of protection/contraception**				
Never	**6.3**	**4.7–7.9**	**6.6**	**4.8–8.2**
Sometimes	**7.9**	**6.8–9**	**7.5**	**6.3–8.7**
Often	2.6	0.9–5.2	**2.5**	**0.9–5.2**
Always	1	-	1	-
**Have you been vaccinated against HPV**				
Yes	**11.2**	**7.1–15.2**	**8.9**	**5.9–13.2**
No	1	-	1	-
**How many doses of HPV vaccine have you received**				
One	1.4	0.7–2.1	1.5	0.7–2.1
two	1.1	0.3–1.9	1.3	0.5–2.2
Three	1	-	1	-

Statistically significant results in **bold**.

## References

[1] Sabeena S., Bhat P.V., Kamath V., Arunkumar G. (2018). Global human papilloma virus vaccine implementation: An update. J. Obstet. Gynaecol. Res..

[2] GLOBOCAN Estimated Cancer Incidence, Mortality and Prevalence Worldwide. http://globocan.iarc.fr/Pages/fact_sheets_cancer.aspx.

[3] Garland S.M., Hernandez-Avila M., Wheeler C.M., Perez G., Harper D.M., Leodolter S., Tang G.W., Ferris D.G., Steben M., Bryan J. (2007). Quadrivalent vaccine against human papillomavirus to prevent anogenital diseases. N. Engl. J. Med..

[4] Hall M.T., Simms K.T., Lew J.B., Smith M.A., Saville M., Canfell K. (2018). Projected future impact of HPV vaccination and primary HPV screening on cervical cancer rates from 2017–2035: Example from Australia. PLoS ONE.

[5] Hariri S., Bennett N.M., Niccolai L.M., Schafer S., Park I.U., Bloch K.C., Unger E.R., Whitney E., Julian P., Scahill M.W. (2015). Reduction in HPV 16/18-associated high grade cervical lesions following HPV vaccine introduction in the United States-2008–2012. Vaccine.

[6] Mesher D., Soldan K., Howell-Jones R., Panwar K., Manyenga P., Jit M., Beddows S., Gill O.N. (2013). Reduction in HPV 16/18 prevalence in sexually active young women following the introduction of HPV immunisation in England. Vaccine.

[7] Garland S.M., Cornall A.M., Brotherton J.M.L., Wark J.D., Malloy M.J., Tabrizi S.N., Group V.S. (2018). Final analysis of a study assessing genital human papillomavirus genoprevalence in young Australian women, following eight years of a national vaccination program. Vaccine.

[8] Wymann M.N., Zographos A.S., Altpeter E., Spicher V.M., Low N., Mausezahl-Feuz M. (2018). Human papillomavirus vaccine uptake in adolescence and adherence to cervical cancer screening in Switzerland: A national cross-sectional survey. Int. J. Public Health.

[9] Jeannot E., Petignat P., Sudre P. (2015). Successful implementation and results of an HPV vaccination program in Geneva Canton, Switzerland. Public Health Rep..

[10] Sundaram N., Duckett K., Yung C.F., Thoon K.C., Sidharta S., Venkatachalam I., Chow A., Yoong J. (2018). “I wouldn’t really believe statistics”—Challenges with influenza vaccine acceptance among healthcare workers in Singapore. Vaccine.

[11] Karafillakis E., Dinca I., Apfel F., Cecconi S., Wurz A., Takacs J., Suk J., Celentano L.P., Kramarz P., Larson H.J. (2016). Vaccine hesitancy among healthcare workers in Europe: A qualitative study. Vaccine.

[12] Ciftci F., Sen E., Demir N., Ciftci O., Erol S., Kayacan O. (2018). Beliefs, attitudes, and activities of healthcare personnel about influenza and pneumococcal vaccines. Hum. Vaccines Immunother..

[13] Osborne S.L., Tabrizi S.N., Brotherton J.M., Cornall A.M., Wark J.D., Wrede C.D., Jayasinghe Y., Gertig D.M., Pitts M.K., Garland S.M. (2015). Assessing genital human papillomavirus genoprevalence in young Australian women following the introduction of a national vaccination program. Vaccine.

[14] Kumakech E., Berggren V., Wabinga H., Lillsunde-Larsson G., Helenius G., Kaliff M., Karlsson M., Kirimunda S., Musubika C., Andersson S. (2016). Significantly Reduced Genoprevalence of Vaccine-Type HPV-16/18 Infections among Vaccinated Compared to Non-Vaccinated Young Women 5.5 Years after a Bivalent HPV-16/18 Vaccine (Cervarix(R)) Pilot Project in Uganda. PLoS ONE.

[15] Machalek D.A., Garland S.M., Brotherton J.M.L., Bateson D., McNamee K., Stewart M., Skinner S.R., Liu B., Cornall A.M., Kaldor J.M. (2018). Very low prevalence of vaccine human papillomavirus (HPV) types among 18 to 35 year old Australian women, nine years following implementation of vaccination. J. Infect. Dis..

[16] Carozzi F., Puliti D., Ocello C., Anastasio P.S., Moliterni E.A., Perinetti E., Serradell L., Burroni E., Confortini M., Mantellini P. (2018). Monitoring vaccine and non-vaccine HPV type prevalence in the post-vaccination era in women living in the Basilicata region, Italy. BMC Infect. Dis..

[17] Woestenberg P.J., King A.J., van der Sande M.A., Donken R., Leussink S., van der Klis F.R., Hoebe C.J., Bogaards J.A., van Benthem B.H. (2017). No evidence for cross-protection of the HPV-16/18 vaccine against HPV-6/11 positivity in female STI clinic visitors. J. Infect..

[18] Markowitz L.E., Liu G., Hariri S., Steinau M., Dunne E.F., Unger E.R. (2016). Prevalence of HPV After Introduction of the Vaccination Program in the United States. Pediatrics.

[19] Saccucci M., Franco E.L., Ding L., Bernstein D.I., Brown D., Kahn J.A. (2018). Non-Vaccine-Type Human Papillomavirus Prevalence After Vaccine Introduction: No Evidence for Type Replacement but Evidence for Cross-Protection. Sex. Transm. Dis..

[20] Mesher D., Soldan K., Lehtinen M., Beddows S., Brisson M., Brotherton J.M., Chow E.P., Cummings T., Drolet M., Fairley C.K. (2016). Population-Level Effects of Human Papillomavirus Vaccination Programs on Infections with Nonvaccine Genotypes. Emerg. Infect. Dis..

[21] Cameron R.L., Kavanagh K., Pan J., Love J., Cuschieri K., Robertson C., Ahmed S., Palmer T., Pollock K.G. (2016). Human Papillomavirus Prevalence and Herd Immunity after Introduction of Vaccination Program, Scotland, 2009–2013. Emerg. Infect. Dis..

[22] Arbyn M., Verdoodt F., Snijders P.J., Verhoef V.M., Suonio E., Dillner L., Minozzi S., Bellisario C., Banzi R., Zhao F.H. (2014). Accuracy of human papillomavirus testing on self-collected versus clinician-collected samples: A meta-analysis. Lancet Oncol..

[23] Nelson E.J., Maynard B.R., Loux T., Fatla J., Gordon R., Arnold L.D. (2017). The acceptability of self-sampled screening for HPV DNA: A systematic review and meta-analysis. Sex. Transm. Infect..

[24] Goggin P., Sauvageau C., Gilca V., Defay F., Lambert G., Mathieu C.S., Guenoun J., Comete E., Coutlee F. (2018). Low prevalence of vaccine-type HPV infections in young women following the implementation of a school-based and catch-up vaccination in Quebec, Canada. Hum. Vaccines Immunother..

[25] Lam J.U.H., Rebolj M., Ejegod D.M., Pedersen H., Rygaard C., Lynge E., Harder E., Thomsen L.T., Kjaer S.K., Bonde J. (2017). Prevalence of Human Papillomavirus in Self-Taken Samples from Screening Nonattenders. J. Clin. Microbiol..

[26] Riesen M., Garcia V., Low N., Althaus C.L. (2017). Modeling the consequences of regional heterogeneity in human papillomavirus (HPV) vaccination uptake on transmission in Switzerland. Vaccine.

[27] Jacot-Guillarmod M., Pasquier J., Greub G., Bongiovanni M., Achtari C., Sahli R. (2017). Impact of HPV vaccination with Gardasil(R) in Switzerland. BMC Infect. Dis..

